# Social Environment and Healthy Investment Behavior: Joint Influence of Culture and Institution on China

**DOI:** 10.3390/ijerph19010607

**Published:** 2022-01-05

**Authors:** Dongao Li, Songdong Shen

**Affiliations:** Business School, Jilin University, Changchun 130012, China; shensd@jlu.edu.cn

**Keywords:** social environment, healthy investment behavior, cultural distance, institutional distance

## Abstract

The influence of the social environment on healthy investment behavior is a vital research topic. This paper focuses on foreign direct investment (FDI) as an important part of its broad impact in improving the level of capital circulation and diversifying the non-systemic risk of a single country portfolio. Using data from 35 countries on direct investment in China, we find that the impact of the social environment on healthy investment behavior is mainly reflected in investors’ resistance to cultural distance and their benefit compensation across institutional distance. In addition, their joint influence is still negative, dominated by cultural distance, which can still verify that institutional distance mitigates the negative effect of cultural distance on FDI. Therefore, in order to promote international healthy investment behavior, it is feasible to improve both the mitigation effect of the institution in the short term and promote the level of cultural exchange in the long term, according to the research results of this paper.

## 1. Introduction

Healthy investment behavior has always been the focus of academic issues. As the basic form of economic growth, the internal capital cycle is universally endogenously restricted by the level of existing knowledge and technology, while foreign capital breaks through this boundary [1]. Existing literature suggests that such exogenous shocks show benefits for both parties, showing attributes of healthy investment behavior. On the one hand, the inflow side gets the positive influence of advanced technology transfer, market demands increase and other improvements of the economy that come with them [2]. On the other hand, the outflow side can open up a larger product market and realize a lower-cost international portfolio [3]. In the context of globalization, international capital that flows fully decentralizes risks among economies, reduces the fluctuation of economic variables in a single economy and contributes to overall economic stability [4]. It will also enhance the efficiency of distribution and make each economy enhance its specialization based on comparative advantages, which benefits the realization of common economic growth. Especially for emerging capital markets, the inflow of foreign capital can mitigate their economic fluctuations, reduce the impact of trade barriers and achieve stable economic growth [5]. More importantly, many studies suggest that FDI has a positive impact on the development of green innovation [6,7,8]. In addition, under strict supervision, FDI can promote the use of clean energy, show more green technology-driven development and reduce the environmental harm of polluting industries. Thus, from the perspective of green and sustainable development, foreign investment has also shown positive effects [9,10].

Because the effects of international capital flow on economic development have been increasingly demonstrated, this paper defines it as healthy investment behavior. As an important path of capital internationalization, foreign direct investment (FDI) has become the core form of healthy investment behavior on account of its substantive and long-term nature. FDI requires investors to own entities overseas and directly engage in production as well as operational activities. Therefore, the demand for additional products and services brought by FDI further stimulates the upstream and downstream of entity enterprises, creating new markets and job opportunities. The changes of the supply chain that arise from the latter as well as forward connections have a positive impact on the formation of industry rules and the establishment of business ecosystems [11,12]. With the entry of foreign capital, some industries reduce production costs and internal financing constraints by deeply embedding themselves in the global value chain (GVC), narrowing the gap with cutting-edge technologies through open development mode [13]. In addition, FDI gains benefits by participating in or controlling the operation of enterprises, which leads to the long-term retention of capital and provides time for the overflow of knowledge. The entry of foreign capital into the industry provides advanced organizational and management experience, reduces the cost of information search and improves the efficiency of research and development. Therefore, domestic enterprises can enhance their innovation ability and improve the innovation speed of the whole industry through “learning through doing”. All the above are reflected in the spillover effect of knowledge [14]. To sum up, international capital inputs are initially reflected in the behavior changes at the micro level, and ultimately leading to the overall efficient development of the macroeconomy, showing a healthier and more sustainable developing trend [15].

However, despite the proven benefits of FDI, its boost is not an easy task, being affected by the social environment, including both soft and hard obstacles. Soft obstacles are mainly reflected in cultural factors, of which the influence on FDI is deeper and more recessive. Culture is defined as “the collective programming of mind” that leads to patterned ways of thinking, feeling and acting which help distinguish “the members of one group or category of people from others” [16]. It forms macro effects through influencing the decision-making process of corporates, such as corporate governance structure, financial policy and risk avoidance level, etc. [17,18,19,20] Hard obstacles are mainly reflected in institutional factors, of which the influence is stronger and more dominant. Institution is defined as a set of tangible and intangible frameworks and rule systems, which is a long-term structural pattern of individual and group behavior [21]. It directly affects the capital flow at the macro, medium and micro levels. This is reflected in the constraint of “legitimacy,” the disadvantage of outsiders in industry access and the cost of information search [22,23].

This paper highlights the possible influence of the social environment on healthy investment behavior. By exploring the independent as well as the joint effect of soft and hard environmental obstacles, the article provides possible solutions to further improve flows of healthy capital. In order to capture this influence more explicitly, the authors chose China as the financing economic entity and other countries with definite investment there as the investing economic ones. The effect is expected to be more meaningful in developing countries, and China, with great availability of samples there to add representativeness, is the largest economy among them. In recent years, China has timely promoted the strategy of attracting foreign investment, which is also worth learning from other developing countries. For example, China has set up five special economic zones in succession, improved policies on attracting foreign investment year by year and gradually reduced restrictive measures on foreign investment. These are embodied in the *Law on Sino-foreign Joint Ventures* and *Catalogue for The Guidance of Foreign Investment Industries*. The data of this research are collected from the World Bank. 

This paper studies the independent and joint effects of cultural distance and institutional distance on foreign investment. We find that the individual–collectivism dimension and the index avoidance dimension of cultural distance have a negative impact on FDI, that is to say, the increase of cultural distance reduces the inflow intention of foreign investment. At the same time, the economic dimension of institutional distance has a positive impact on FDI, that is to say, the increase of institutional distance improves the inflow willingness of foreign capital. The reasons may be as follows. Expected returns after overcoming institutional obstacles are conducive to risk compensation because of its higher predictability, which may appeal to foreign capital. In comparison, it is more difficult to overcome its negative impact of cultural obstacles to achieve excess returns due to its lower predictability, which may lead to the reduction of foreign-funded projects. Furthermore, we examine the combined effect of cultural distance and institutional distance and find that institutional distance can mitigate the negative relationship between cultural distance and foreign investment, but still cannot offset the negative impact of cultural distance. It is probably a combined result of two factors: The effect of cultural distance is deeper and more significant, while the risk compensation brought by crossing institutional distance cannot cover the potential risk cost of crossing cultural distance. These suggest the social environment’s important influence on FDI is worthy of academic and practical attention.

This article may contribute to theory and practice. In terms of theory, in the issue of the relationship between environmental barriers and foreign investment, this paper innovatively proposes two dimensions: soft environment barriers and hard environment barriers. Through theoretical deduction and empirical results, it is preliminarily confirmed that soft environmental barriers and hard environmental barriers have different impacts on foreign capital inflows in terms of time and depth, and the research conclusions provide a reference for relevant theory. At the practical level, the effect of institutional distance is earlier and more surmountable, while the influence of cultural distance is later and less predictable. Taking into account the important impact of foreign capital inflows on the sustainable development of the domestic economy, we make recommendations from both cultural and institutional perspectives. Developing countries can offer preferential institutional policies to attract foreign investment in the early stages while sticking to their own advantageous institutional strategies in the long run. However, the attention to culture must be persisted because the effects of cultural exchange can be only reflected after plenty of effort.

The remainder of the paper is as follows. Section 2 provides an overview of materials and methods. Section 3 represents research results and analysis. Section 4 reports the discussion, and Section 5 reports the conclusion.

## 2. Materials and Methods

### 2.1. Literature Review and Research Hypotheses

Culture measures informal drivers of an economy and the social value system within society [24]. With more and more research on cultural environment and foreign investment, the concept of cultural distance is proposed to measure the cultural differences between two economies. In organizational studies, the cultural distance between countries is usually defined in terms of differences between stable value systems, which is a broader concept of differences such as traditions, norms, customs and so on [25,26]. Cultural distance refers to the degree of cultural difference between various societies, regions and countries, as the values and belief systems embedded in a society influence each individual’s performance [27].

The most common measurement of cultural distance was established by Hofstede [24]. For its construction, Hofstede collected data from 116,000 surveys in 20 languages from 40 countries on attitudes and values, ranging from workers to PhDs and top managers. Because its data construction process is the most complete in this issue so far and the evolution of culture is relatively slow and long-term, the data can be used for a long time by many articles on economics and management [28]. Four cultural values were created according to the data of cultural concepts. They can be divided into power distance, which weighs the recognition degree of employees in different countries when they encounter unfair rights; collectivism–individualism, which is used as the measuring standard for the supremacy of collective interests or individual interests in different countries; the avoidance of uncertainty, weighing the tolerance of each country when the risk is vague; and masculinity–femininity, weighing the recognition degree of material success or social harmony and stability in different cultures—the male-dominated society values the realization of life value more, while the female-dominated society values interpersonal harmony and happiness index more. Although there are many ways to measure cultural distance, this definition is still the most widely applied one at present, and this definition is also followed in this paper. 

Culture is an intrinsic, relatively stable and implicit factor in the social environment, with its complicated core. Therefore, culture has a complex and multi-level influence relationship on the cross-border circulation of capital [29]. In general, when transnational capital enters foreign markets, it is exposed to different social conventions and implicit assumptions, which may seem unfamiliar, vague, challenging and require adjustment and adaptation [30]. High cultural distance will magnify the uncertainty and risk perceived by transnational capital and affect the effective transfer of its internal foundation, process and management experience to the recipient country. Due to differences in culture-induced attitudes, beliefs, value systems and behavioral assumptions, a high cultural distance may increase the challenges for MNC to successfully establish, monitor and maintain relationships. In contrast, low cultural distance significantly alleviates these challenges [31].

First, cultural distance can lead to cognitive impairment, which is mainly reflected in the process of information exchange between transnational investment projects at various levels after landing. This information interaction includes the government, competitors, supply chain enterprises, employees and so on. The degree of cognitive impairment will hinder the efficiency of knowledge transfer and the effectiveness of feedback, especially the tacit knowledge most relevant to enterprise operation. Whether such knowledge can be replicated is directly related to whether cross-border capital can achieve the expected income level. At the same time, the accumulation of cognitive impairment may also lead to conflict or even confrontation, leading to a reduction in business performance. Therefore, from this perspective, the greater the cultural distance, the less attractive the host country is to cross-border capital investment [32,33]. Second, cultural distance can lead to organizational and management barriers. Cross-border capital investment means the development of cross-border branch business. Cultural differences may increase mutual distrust, and the lack of trust reduces the degree of mutual support, which intensifies the hidden costs in the principal-agent relationship. With the increasing imperceptibility of agency issues, principals need to face greater information collection costs and information acquisition pressure in order to reduce agency costs, which also reduces the enthusiasm of cross-border capital inflows [34]. Thirdly, cultural distance will lead to differences in consumer preferences, and the change in demand preferences puts forward high requirements for strategic adjustment, which increases the input cost of cross-border capital. In addition, strategic adjustments due to cultural preferences also mean two-way coordination of investments in the medium term, which brings greater risk. The increase in costs and risks caused by consumer preference will also increase the difficulty of cross-border investment [35].

At the same time, some articles believe that cultural distance provides potential power for knowledge collision brought by international capital flow. Differences in knowledge reserves, organizational procedures and management mechanisms may bring complementary effects and ultimately form differentiated advantages [36]. However, overall, culture distance is the main obstacle of a soft environment, bringing about hidden capital leakage. Its impact on international investment has high unpredictability, and late differentiation advantage attractiveness is difficult to make up for the upfront costs and risks. Thus, this paper argues that multiple effects still end negatively. Considering that the four dimensions of cultural distance may show different ways of influence in the same research topic, Hypothesis 1 is proposed as follows, and the possible differences of influence effects of different dimensions are explored in the empirical results:

**Hypothesis** **1** **(H1).** *Cultural distance has a negative impact on foreign direct investment*.

Institutional theory holds that an organization needs to follow certain conventions to achieve the legitimacy of its existence. North defines institution as the game rules of a society and, more strictly speaking, artificial constraints set to determine people’s relationship with each other, including regulative, normative and cognitive constraints [37]. This definition has been followed and continued. According to institutional theory, organizations are embedded in a large institutional environment, which directly affects the transaction costs of enterprises and then determines the risks and benefits of economic activities [38]. The theory emphasizes that enterprises need to obey the local system and be recognized by the local rule system to survive and develop [39]. As the practices, policies and structures of enterprises need to comply with the requirements of the institutional environment, the influence of institutional environment obstacle on foreign direct investment is mainly reflected in the level of “legitimacy disadvantage” faced by transnational capital when it enters the real market.

The institutional difference between the two economies is defined as institutional distance. Foreign investment faces difficulties and incurs additional costs due to political and economic risks in the host country, which is reflected in the political dimension and economic dimension of institutional distance [40]. The identity label of “outsiders” formed by cross-border capital inflows leads to disadvantages in obtaining local recognition, namely “legitimacy” disadvantage [41]. The degree of acceptance or rejection of transnational capital is different with different institutional distances. The higher the institutional distance is, the more difficult it is for cross-border acquisition to be recognized by local residents or governments, the more likely the acquirer will be rejected and the more likely cross-border acquisition will be interrupted. Similarly, the establishment of multinational enterprises will also face the cost of information collection and relationship establishment of a higher degree due to institutional differences, which are manifested as obvious capital friction. The disadvantage of “legitimacy” is also manifested within the organization. Institutional distance will affect the difficulty for overseas branches to obtain internal legitimacy in the head office, thus increasing coordination between departments and cost control [42]. It is of great significance to overcome institutional differences in the realization of investment objectives in transnational investment [43].

However, with the increase of the number of FDI, the negative impact of institutional distance began to ease, and the “outsider advantage” gradually emerged [44]. Existing studies show that previous M&A experience has a critical impact on subsequent M&A activities through knowledge transfer, and successful cross-border M&A experience in international business services can overcome the impact of institutional distance and improve the completion efficiency [45]. The unfamiliar institutional environment, while increasing uncertainty and risk, can often stimulate the acquirer to make use of the positive role of past experience through conscious and active learning [46].

At the same time, different from the cultural environment, the barriers to entry connected with the institutional environment are more reflected in the explicit level and the early stage of operation. Therefore, the additional costs of institutional differences are often predicted in advance, which reduces the risk of capital landing. With the accumulation of organizational learning and internationalization experience, many institutional rules can be coded and quickly familiarized, and the negative impact of institutional differences on the inflow of foreign capital will gradually weaken and even be deflected [47]. When the effect of differentiation advantage is greater than the effect of legitimacy disadvantage, the influence of institutional distance on capital gains will be deflected, which is reflected in “first-mover welfare”. This is the spillover economic compensation of foreign capital after breaking through the original institutional barriers, and the capital premium it brings can offset earlier flow friction, which makes it more attractive to foreign capital. 

In addition, existing literature shows that when the degree of tacit knowledge is reduced, it will be less and less difficult to replicate tacit knowledge across borders [48]. Compared with cultural distance, institutional distance is easier to capture and less hidden. At the same time, China’s restrictions on FDI are decreasing year by year, which also reduces the difficulty for transnational capital to overcome institutional distance and gain profits. Specifically, it can be verified through *Law on Sino-Foreign Joint Ventures* and *Catalogue for The Guidance of Foreign Investment Industries*.

Considering the path for enterprises to overcome institutional distance and obtain excess returns, existing studies have proposed External Mimicry and Internal Mimicry [49,50,51]. The former refers to the success of foreign capital in opening similar markets abroad. The latter refers to foreign capital deciding how to set up overseas branches based on its gradually accumulated experience. For example, a successful experience in a host country is likely to be repeated by the parent company in other similar regions. Based on the overall environment of perfect laws and regulations and deregulation in China, we suggest the advantages of overcoming institutional distance brought by enterprises are likely to exceed the disadvantages of outsiders. Thus, Hypothesis 2 is proposed as follows:

**Hypothesis** **2** **(H2).** *Institutional distance has a positive impact on foreign direct investment*.

FDI is transnational investment behavior at the enterprise level. Existing studies have decomposed the capital input and earnings process of multinational enterprises into two parts: location choice and entry mode and operation integration and revenue realization [52]. The implicit influence of cultural distance as a soft environmental obstacle and the explicit influence of institutional distance as a hard environmental obstacle show different forms in different stages.

In the first stage of location choice and entry mode, foreign capital needs to weigh the benefits brought by low labor price and potential market size against the costs brought by overcoming environmental barriers to decide the country to be invested in [53]. In this process, the cost of institutional distance, as a hard environmental barrier, shows higher observability and predictability. At the same time, cultural distance, as a soft environmental barrier, has an unpredictable cost at this point, for it can only be reflected in the specific organization and management of the company [54]. Therefore, the economic model designed by foreign capital at the initial stage of entry contains more institutional distance costs (compared to cultural distance) and still choosing to invest means that foreign capital is prepared to overcome institutional distance [55,56].

In the second stage of operation integration and revenue realization, foreign capital needs to work out how to manage corporate operations. This includes putting in place appropriate corporate governance arrangements, achieving the best possible integration, coordination and control and minimizing the cost of replicating knowledge, technology and management expertise [57]. At this stage, the cost of the political and economic differences implied by institutional distance becomes more transparent as the specific location of industries and firms is determined. In contrast, the incalculable cost of cultural distance is gradually reflected: contract incompleteness caused by information asymmetry, work efficiency decline caused by a continuous cultural conflict of employees, low ability to resist crisis caused by low organizational harmony, etc. [58,59]

Based on the analysis of the specific stage of foreign capital establishing companies and realizing profits, we find that institutional distance and cultural distance have different stages and internal mechanisms of influence on FDI. The cost of institutional distance appears earlier, is more explicit and is relatively better to predict, while the cost of cultural distance appears later, is more implicit and is relatively difficult to predict. Therefore, after a long period of multiple games, the influence of institutional distance on FDI is more likely to be expressed as excess returns after obstacle crossing, while the influence of cultural distance on FDI is more likely to be manifested as a conflict caused by uncertainty and unpredictability. 

Furthermore, after multiple games between transnational capital and environmental barriers, the ability of foreign capital to cross-institutional distance gradually increases, so the uncertainty risk caused by institutional distance decreases, which means that the total risk faced by transnational investment decreases. That also signifies the return per unit across institutional distance is higher. In general, under the circumstance of lower risks and higher returns, there will be more attempts of transnational capital [60,61]. Therefore, we speculate that with the increase of institutional distance, the negative impact of cultural distance on FDI will also weaken.

Therefore, we conclude that institutional distance can mitigate the negative relationship between cultural distance and foreign investment. However, because cultural implantation is more implicit and in depth, the spillover economic compensation caused by breaking institutional distance can hardly fully offset the cost caused by cultural distance [62]. Thus, this moderating effect is relatively weak, and Hypothesis 3 is proposed as follows:

**Hypothesis** **3** **(H3).** *The joint influence of cultural distance and institutional distance on foreign direct investment is negative*.

### 2.2. Sample Selection

In order to verify the above assumptions, this paper selects China as the country to be invested in and other countries that have a direct investment in China as the countries that invest abroad. Because China has the largest emerging capital market, as a good representation for developing countries, the research conclusion can provide a reference for other developing economies. Meanwhile, Hong Kong, Macao and Taiwan are special administrative regions of China, with their economic and political policies different from those of the mainland in some ways. Thus, the data in this paper only include that of the Chinese mainland. A total of 420 observations from 35 countries and regions are selected for data integrity and availability. These countries or regions include Argentina, Austria, Belgium, Brazil, Canada, Chile, Colombia, Denmark, Ecuador, Finland, France, Greece, Hong Kong, India, Indonesia, Ireland, Israel, Italy, Japan, South Korea, Malaysia, Mexico, Netherlands, New Zealand, Norway, Pakistan, Philippines, Portugal, Singapore, Sweden, Thailand, Turkey, US, Uruguay and Venezuela. The data is mainly from the official website of the International Monetary Fund, with a time period from 2008 to 2019.

### 2.3. Variable Definitions

The explained variable of the article is TPI, the proportion of one other country’s investment in China divided by its total portfolio investment. Compared with absolute value, relative value can better reflect the difference of investment intention, more clearly to capture whether environmental obstacles have an impact on foreign direct investment. Explanatory variables include two dimensions of social environment obstacles, which are cultural distance and institutional distance. 

The measurement of culture environmental distance is based on Hofstede’s culture dimensions, which is consistent with previous studies [63,64,65]. It has made outstanding contributions to cross-cultural studies, mainly including four dimensions: power distance (PDI), individualism–collectivism (IDV), uncertainty avoidance (UAI) and masculinity–femininity (MAS). The values of the four dimensions are between 0 and 100, and the higher the value is, the bigger the cultural distance is. 

The measurement of institutional environment is divided into two dimensions referring to the existing literature: political environment measured as WGI index in the world bank and economic environment measured as the heritage foundation’s index of EFI [66,67]. The calculation of WGI is mainly at the political level through official calculation, based on the index value of corruption, government effectiveness, political stability, regulatory quality, rule of law and voice and accountability. The calculation of EFI is mainly at the economic level through official calculation, based on the index value of business freedom, freer trade, fiscal freedom, monetary freedom, black market freedom and financial freedom, labor freedom, freedom corruption, government spending and property rights protection. The values of WGI range from 0 to 100, while that of EFI range from 2.5 to 2.5, where a higher score means a better institutional environment. It is worth noting that although China’s scores in both indexes are a little lower thathe n middle in a group of about 200 countries, they have been rising increasingly since 2008. Following the general practice of existing papers, we define the absolute value of the difference between the institutional environment scores of two economies as the institutional distance [68]. Therefore, WGId is the proxy variable of the political institutional distance, measured by the absolute value of the score difference of the political environment. In the same way, EFId is the proxy variable of the economic institutional distance, measured by the absolute value of the score difference of the economic environment.

As the basic model applied in this paper is the gravity model, we follow the control variables in previous papers about foreign direct investment by gravity model [69]. GDP (gross domestic product) represents the gravity of the investing countries, CGDP represents China’s GDP, and GIST (geographic distance) represents the repulsive force between the two countries. Then, we refer to the practices of relevant literature and introduce the following dummy variables [70,71]. Comlang refers to whether the investor and investee share a common official language; if they have a common language, the variable takes 1, otherwise, it takes 0. Comrelig refers to whether the investor and investee have common religious beliefs; if they have one or more common religious beliefs, the variable takes 1, otherwise, it takes 0. Comleg refers to whether the investor and investee have a common legal source; if they have a common legal source, the variable takes 1, otherwise takes 0. Contig refers to whether the investor and investee share a common border, an important variable to measure the distance between the importance and information cost; if they share one, the variable takes 1, otherwise, it takes 0. In addition, according to the theory of law and finance, we also add two control variables of investor institution: Inves (investor protection intensity index) and Judi (legal system effectiveness index). Finally, this paper controls for annual fixed effects to mitigate the annual macro environment from interfering with the results. The specific variable definitions are in Table 1.

### 2.4. The Model 

The gravity model was created in the 1980s, with the idea that the geographical distance between two economies would affect their economic and trade interactions. As the model has since evolved, the distance between the two economies has widened in other ways. The practical operation of investment research using a gravity model is to assume that the mean value of other unobservable factors is 0. Then the gravity model and estimation method are established according to the research object to measure the theoretical investment frontier.

The traditional frontier gravity model is:

(1) $$FDI_{ijt}=f\left(X_{ijt},\beta \right)$$

In the above formula, *i* represents China, *j* represents the investor country, *t* represents the year and *FDI_ijt_* represents the amount of direct investment China received from different countries in *t* year. *X_ijt_* represents the influencing factors of *FDI_ijt_* and the coefficient of variables to be estimated. Then, the environmental obstacle term is taken into account, as the interpretation of investment resistance. The new equation is as follows:

(2) $$FDI_{ijt}=f\left(X_{ijt},\beta \right)exp\left(-U_{ijt}\right)$$

where *U_ijt_* is the environmental resistance term, that is, factors that may lead to reduced investment. Therefore, *E_ijt_* = *exp*(−*U_ij_*_t_), as the impact of environmental resistance on FDI. When *U_ijt_* ≥ 0, 0 < *E_ijt_* < 1, and *E_ijt_* reflects the environmental multiplier of the foreign direct investment attracted by country *i* to country *j* in period *t*.

The closer *U_ijt_* is to 0, the closer *E_ijt_* is to 1, reflecting that the foreign direct investment of country *i* to country *j* in period *t* is less and less affected by environmental factors. Further, the random perturbation term is introduced on the right side of the equation, and the new production function is:

(3) $$FDI_{ijt}=f\left(X_{ijt},\beta \right)exp\left(L_{ijt}-U_{ijt}\right)$$

Take the natural logarithm of both sides of this function, and it becomes:

(4) $$lnFDI_{ijt}=lnf\left(X_{ijt},\beta \right)+L_{ijt}-U_{ijt}$$

where *U_ijt_* can be decomposed into *U_ijt_* = *Z_ijt_* + *W_ijt_*+*α_ijt_*, with *Z_ijt_* representing soft environment resistance and *W_ijt_* representing hard environment resistance. *α_ijt_* is the random perturbation term. We substitute it into the latest to obtain the stochastic frontier function:

(5) $$lnFDI_{ijt}=lnf\left(X_{ijt},\beta \right)-Z_{ijt}-W_{ijt}-\alpha_{ijt}+L_{ijt}$$

On the basis of the above, the stochastic frontier model of inefficient terms proposed is brought into the model. At the same time, referring to the experience of previous literature, combined with variable selection in this paper, we set regression models for the hypotheses mentioned above.

Firstly, to test the impact of cultural environment obstacles on foreign direct investment (Hypothesis 1), we estimate the following model:

(6) $$\begin{array}{ll} lnTPI_{ijt}=\partial_{0}+ & \partial_{1}lnPDI_{ijt}+\partial_{2}lnIDV_{ijt}+\partial_{3}lnUAI_{ijt}\\ & +\partial_{4}lnMAS_{ijt}+\partial_{5}lnCGDP_{ijt}+\partial_{6}lnGDP_{ijt}+\partial_{7}lnGIST_{ijt}\\ & +\partial_{8}Comlang_{ijt}+\partial_{9}Comrelig_{ijt}+\partial_{10}Comleg_{ijt}\\ & +\partial_{11}Contig_{ijt}+Time+\varepsilon_{ijt} \end{array}$$

*TPI_ijt_* represents the investment from one country to China divided by its total investment. Compared with absolute value, relative value can more clearly reflect the investment intention of the investing country. *PDI_ijt_*, *IDV_ijt_*, *UAI_ijt_* and *MAS_ijt_* are four dimensions of cultural distance at the country level. *PDI_ijt_* represents the level of power distance, *IDV_ijt_* represents the level of individualism distance, *UAI_ijt_* represents the level of uncertainty avoidance and *MAS_ijt_* represents the level of masculinity distance. The macroeconomic control variables consist of *CGDP_ijt_* (the natural logarithm of China’s GDP), *GDP_ijt_* (the natural logarithm of the GDP of the investing country) and *GIST_ijt_* (the natural logarithm of the geographic distance between investing country and China). *Comlang_ijt_* is a dummy variable that represents whether the investing country has a common language with China. *Comrelig_ijt_* is a dummy variable that represents whether the investing country has a common religion with China. *Comleg_ijt_* is a dummy variable that represents whether the investing country has a common legal system with China. Lastly, *Contig_ijt_* is a dummy variable that represents whether the investing country has a common border with China. Time represents the year dummy variable.

Then, to test the impact of institutional environment obstacles on foreign direct investment (Hypothesis 2), we estimate the following model:

(7) $$\begin{array}{ll} lnTPI_{ijt}=\partial_{0}+ & \partial_{1}lnWGId_{ijt}+\partial_{2}lnEFId_{ijt}+\partial_{3}lnCGDP_{ijt}+\partial_{4}lnGDP_{ijt}\\ & +\partial_{5}lnGIST_{ijt}+\partial_{6}Inves_{ijt}+\partial_{7}Judi_{ijt}+\partial_{8}Contig_{ijt}\\ & +Time+\varepsilon_{ijt} \end{array}$$

*WGId_ijt_* and *EFId_ijt_* are two dimensions of institutional distance at the country level. *WGId_ijt_* represents the level of political distance from the investing country to China. First, the two economies’ distance is measured according to corruption, government effectiveness, political stability, regulatory quality, rule of law, voice and accountability, of which their scores are calculated (full marks are 100 points). Ultimately, the value of *WGId_ijt_* is the average value of these aspects. *EFId_ijt_* represents the level of economic institutional distance from the investing country to China. First, the two economies’ distance is measured according to business freedom, freer trade, fiscal freedom, monetary freedom, black market freedom and financial freedom, labor freedom, freedom corruption, government spending and property rights protection, of which their scores are calculated (full marks are 100 points). Ultimately, the value of *EFId_ijt_* is the average value of these aspects. Other control variables not mentioned above are *Inves_ijt_*, which represents the investor protection intensity index, and *Judi_ijt_*, which represents the legal system effectiveness index.

Third, to test the joint effect of culture environment obstacle and institution environment obstacle on foreign direct investment (Hypothesis 3), based on the experience of previous articles, this paper synthesizes the four dimensions of cultural distance as an overall index. Though the measurement of cultural institutions in Hypothesis 1 is accepted by most papers, we must admit that this measurement method has its shortcomings. As Hofstede index only has values of different dimensions and does not use statistical methods to synthesize a comprehensive index, it is only suitable for measuring the details of cultural distance and cannot be used to judge the comprehensive index of that. Therefore, in the following hypothesis, we use the composite index calculated based on this. There are two main research methods in previous articles. One is the KS index calculated by the compound index method, and the other one is the EU index calculated by the Euclidean distance method [72]. The specific calculation formula is as follows:

$$\mathrm{KS}\;\mathrm{index}:\;CD_{ic}=\sum_{j=1}^{4}\left[{\left(D_{ji}-D_{jc}\right)}^{2}/V_{j}\right]/4\;\mathrm{EU}\;\mathrm{index}:\;CD_{ic}=\sqrt{\sum_{j=1}^{4}\left[{\left(D_{ji}-D_{jc}\right)}^{2}/V_{j}\right]}$$

In the formula, *CD_ic_* refers to a composite index of cultural distance between one other country and China. *D_ji_* represents the cultural dimension *j*’s score of country *i*; *V_j_* represents the variance of cultural dimension *j*. The lower the index is, the smaller are the cultural differences between the investing country and China and vice versa. Thus, in order to test Hypothesis 3, we estimate the following model:

(8) $$\begin{array}{ll} lnTPI_{ijt}=\partial_{0}+ & \partial_{1}lnCuI_{ijt}+\partial_{2}lnWGI_{ijt}+\partial_{3}lnEFI_{ijt}\\ & +\partial_{4}lnCul_{ijt}*lnWGI_{ijt}+\partial_{5}lnCul_{ijt}*lnEFI_{ijt}+\partial_{6}lnCGDP_{ijt}\\ & +\partial_{7}lnGDP_{ijt}+\partial_{8}lnGIST_{ijt}+\partial_{9}Inves_{ijt}+\partial_{10}Judi_{ijt}\\ & +\partial_{11}Contig_{ijt}+\partial_{12}Comlang_{ijt}+\partial_{9}Comrelig_{ijt}\\ & +\partial_{10}Comleg_{ijt}+Time+\varepsilon_{ijt}lnTPI_{ijt}\\ & =\partial_{0}+\partial_{1}lnWGId_{ijt}+\partial_{2}lnEFId_{ijt}+\partial_{3}lnCGDP_{ijt}\\ & +\partial_{4}lnGDP_{ijt}+\partial_{5}lnGIST_{ijt}+\partial_{6}lnves_{ijt}+\partial_{7}Judi_{ijt}\\ & +\partial_{8}Contig_{ijt}+Time+\varepsilon_{ijt} \end{array}$$

*Cul_ijt_* refers to the composite index of cultural distance between one other country and China. The KS index and EU index were substituted into the regression equation as *Cul_ijt_* to enhance the robustness of the results.

## 3. Results

### 3.1. Descriptive Statistics

Summary statistics of the sample data are presented in Table 2. It can be seen that the investment level of each country is discrete, and the average value is not high, which indicates the proportion of the capital flow to the developing countries still needs more improvement. Therefore, it is of practical significance to study foreign direct investment in developing countries. Among the four dimensions of cultural distance, IDV and UAI reflect large country differences, while MAS reflects small country differences. In the data of the two dimensions of institutional distance, it is obvious that WGI has a lower degree of dispersion while EFI has a higher one.

**Figure 1:**
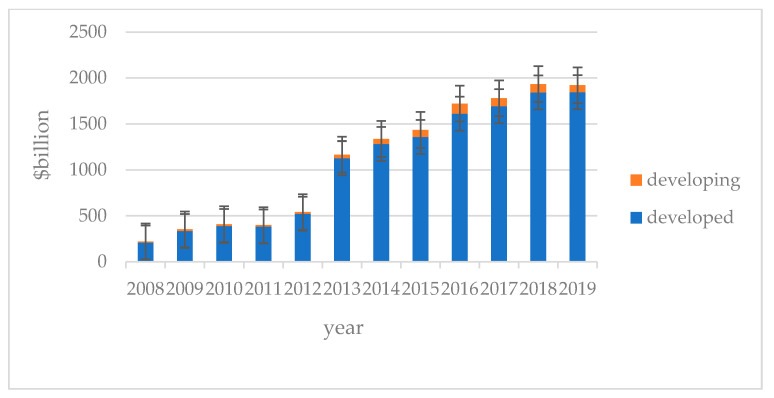
The trend of foreign direct investment.

Table 3 reports the amount of direct investment into China by other countries or regions from 2008 to 2019, and then Figure 1 provides a more intuitive histogram of the data there. It can be seen that the amount of foreign direct investment in China has been increasing during the data period. Among them, the proportion of investment in developed countries or regions is significantly higher than that in developing countries, which is consistent with the theoretical deduction mentioned above.

### 3.2. Main Empirical Results

In order to verify the influence of the social environment on foreign direct investment, we conduct regression analysis on Model 1, Model 2 and Model 3. The results are shown in Table 4.

Table 4, Column 2 reports the estimation results of Model 1, exploring the influence of cultural distance on foreign investment. We find a negative association between the two dimensions of cultural distance and foreign investment. A one-standard-deviation decrease in *IDV* is associated with a higher *TPI* of 0.172 bps, significant at the 5% level. In the same way, a one-standard-deviation decrease in *UAI* is associated with a higher *TPI* of 0.152 bps, significant at the 5% level. At the same time, the empirical results also show that the other two dimensions of cultural distance, *PDI* and *MAS*, do not show a significant correlation with *TPI* in our model test. It is also consistent with the theoretical deduction above, showing that the effect of different dimensions may be reflected in different economic events.

Table 4, Column 3 reports the estimation results of Model 2, exploring the influence of institutional distance on foreign investment. We find a positive association between the economic dimension of institutional distance and foreign investment. From an economic standpoint, a one-standard-deviation increase in *EFId* is associated with a higher *TPI* of 0.304 bps, significant at the 1% level. However, the empirical results also show *WGId*, the political dimension of institutional distance, do not show a significant correlation with *TPI* in our model test. This may be due to the fact that, in the case of sound laws, differences in political systems do not significantly promote or hinder foreign economic inflows, while the difference in economic systems may bring arbitrage opportunities.

Table 4, Columns 4 and 5 report the estimation results of Model 3, exploring the joint influence of cultural distance and institutional distance on foreign investment. The KS index is used for measuring *Cul* in Column 4, while the EU index is used for measuring *Cul* in Column 5. It shows that the joint coefficient of *Cul* and *EFId* is significantly negative, as the positive effect of institutional distance on *FDI* cannot offset the negative one of cultural distance, consistent with Hypothesis 3 (H3). At the same time, the estimated interaction coefficient between *Cul* and *WGId* is also negative but not statistically significant. The difference between the results of the joint influence further verifies the different economic consequences of the two institutional distance dimensions. The consequences of the political dimension are not obvious in foreign investment, while the consequences of the economic dimension significantly affect foreign investment itself and also moderate the relationship between cultural distance and foreign investment.

### 3.3. Robustness Test Results

Existing articles show that FDI is mainly embodied in two specific forms: greenfield investment (including wholly-owned and joint investment) and cross-border M&A [73,74,75]. The above test is mainly based on the total amount of foreign investment, omitting the distinction between different forms of it, which may conceal some details. Therefore, we will further examine the influence of cultural distance and institutional distance on heterogeneous ways of foreign investment. The data comes from United Nations Conference on Trade and Development (UNCTAD), including the same 35 countries. Further empirical regression results are in Table 5.

It is shown in Table 5 that when FDI is divided into greenfield investment and cross-border M&A, the interaction term between cultural distance and *WGId* is not significant, and the interaction term between *Cul* and *EFId* is still significantly negative. It is worth pointing out that the influence of cultural distance, institutional distance and their interaction is greater in the group of cross-border M&A. This may be due to the higher environmental barriers that cross-border M&A need to overcome, as this type of entry involves a larger one-time investment amount. Greenfield investment, by contrast, is a constant infusion of investment, which gives companies a cushion.

## 4. Discussion

Healthy investment behavior among countries is the guarantee of stable global economic growth. Different from the mutual transfer of capital between developed countries, developing countries have a larger capital gap, more technical barriers to overcome, and a more imperfect industrial environment. As a result, developing countries have a higher demand for foreign investment, and hindrance factors affecting capital supply are worth studying. This article highlights the social-environmental impact, embodied in cultural distance and system distance.

For cultural distance, we consider four dimensions, namely, power distance, individualism–collectivism, uncertainty avoidance and masculinity–femininity. The results show that power distance and masculinity–femininity are unrelated to FDI, while individualism–collectivism and uncertainty avoidance have negative effects on FDI. This may be because power distance and masculinity–femininity do not significantly affect the cognitive or management barriers after foreign capital landing. By contrast, individualism–collectivism has an effect on interpersonal communication between organizations and mutual support after capital crosses borders. Furthermore, uncertainty avoidance may lead to cognitive impairment and consumer resistance at the same time, which also reduces the propensity of foreign investors to enter.

For institutional distance, we consider the political dimension and economic dimension. It shows that economic distance has a significant positive influence on FDI, while political distance has no significant influence on FDI. It is possible that China’s policies towards capital inflows have relatively stabilized, and thus, the effect of political distance has not been observed significantly. At the same time, the impact of economic distance is more of a market access threshold. When foreign capital can cross this barrier, it may get more capital return, which is a great attraction.

When examining the joint effect of cultural distance and institutional distance, we find that the results are still negative. This is consistent with theoretical deduction, indicating that the culture effect plays a larger part in the interaction.

## 5. Conclusions

Taking China as the research subject, this paper analyzes how social environmental factors affect foreign investment across countries. In this topic, we mainly study the independent effect and joint influence of cultural distance as a soft social environment obstacle and institutional distance as a hard social environment obstacle.

It shows that cultural distance has a negative effect on FDI, which is likely due to the fact that cultural distance hinders knowledge transfer, organizational communication and consumption preference change, thus reducing the attraction to foreign direct investment. At the same time, institutional distance shows a positive impact on FDI, likely to result from the extra income generated by the entry barrier of the economic system environment being crossed, and the compensation for risk is evaluated as profitable by foreign capital.

Last but not least, the economic compensation effect caused by institutional distance cannot offset the capital leakage caused by cultural distance. This may be due to the fact that the influence of the culture as a society’s overall ideology is more fundamental. However, it is not immutable. From the perspective of the social environment, we propose temporal heterogeneity recommendations on this issue. In the long term, a sustainable program that promotes cultural exchange and dissemination is effective as this will reduce the risk of capital in crossing borders. In the short term, it is essential to play the role of institutions to offset the potential risks of cultural barriers, such as more specific policies to attract investment, more transparent market rules, better investor protection regulations and so on. The joint mechanism of cultural environment and institutional environment will contribute to the development of transnational healthy investment behavior.

## Figures and Tables

**Table 1:** Definition of variables.

Variable	Definition	Source
TPI	The country’s investment in China divided by its total portfolio investment	The International Monetary Fund website (IMF)
PDI	Hofstede’s cultural index on power distance.	Hofstede (2001)
IDV	Hofstede’s cultural index on individualism.	Hofstede (2001)
UAI	Hofstede’s cultural index on uncertainty avoidance.	Hofstede (2001)
MAS	Hofstede’s cultural index on masculinity.	Hofstede (2001)
WGId	A composite index calculated based on corruption, government effectiveness, political stability, regulatory quality, rule of law and voice & accountability.	IMF
EFId	A composite index calculated based on business freedom, freer trade, fiscal freedom, monetary freedom, black market freedom and financial freedom, labor freedom, freedom corruption, government spending, property rights protection.	IMF
CGDP	China’s Gross Domestic Product value	The World Bank
GDP	Investing country’s Gross Domestic Product value	The World Bank
GDIST	The capital of the distance between the two countries	CEPII database
Comlang	If the two countries have a common official language, the value is 1, otherwise, it is 0	CEPII database
Comrelig	If the two countries have a common religion, the value is 1, otherwise, it is 0	CIA world Factbook
Comleg	If the two countries have a common legal system, the value is 1, otherwise, it is 0	LLSV Law and finance
Contig	If the two countries have a common border, the value is 1, otherwise, it is 0	CEPII database
INVES	The investor protection index	The World Bank
JUDI	Investor’s legal effectiveness index system	LLSV Law and finance

**Table 2:** Summary statistics of sample data.

Variable	Mean	Median	Std
TPI	0.018	0.00158	0.0478
IDV	44.33	37.50	24.58
PDI	56.47	62.00	22.53
UAI	63.83	68.50	25.28
MAS	49.44	51.00	19.70
WGId	0.635	0.76	0.93
EFId	66.87	68.05	11.26
GDP	1.4 × 10^12^	3.8 × 10^11^	2.99 × 10^12^
GDIST	8678.09	7623.44	5005.00
Comrelig	0.69	1.00	0.464
Comleg	0.09	0.00	0.28
Comlang	0.09	0.00	0.28
Contig	0.09	0.00	0.28
Inves	6.45	6.50	1.17
Judi	7.66	8.00	2.21

Note: Table 2 presents the descriptive statistics for our regression variables. The sample comprises 420 observations from 35 countries and regions for the period 2008–2019.

**Table 3:** The trend of foreign direct investment.

Time	Total Investment	Developed	Developing
2008	221.504	208.622	12.915
2009	353.085	334.225	18.904
2010	409.115	388.854	20.285
2011	399.529	382.726	16.825
2012	541.850	522.601	19.272
2013	1166.189	1127.661	38.575
2014	1336.321	1281.488	54.881
2015	1434.670	1357.707	77.010
2016	1720.085	1610.050	110.035
2017	1779.064	1692.241	86.823
2018	1933.381	1842.138	91.243
2019	1920.721	1845.599	75.122

Note: Unit is US dollars, billions.

**Table 4:** The empirical results.

Variable	Model 1	Model 2	Model 3 (KS)	Model 3 (EU)
Ln(1+PDI)	−0.001			
Ln(1+IDV)	−0.007 **			
Ln(1+UAI)	−0.006 **			
Ln(1+MAS)	−0.001			
Ln(1+Cul)			0.061 ***	0.091 ***
Ln(1+WGId)		−0.011	0.042 *	0.111 **
Ln(1+EFId)		0.027 ***	0.047 ***	0.084 ***
Ln(1+Cul) * Ln(1+WGId)			−0.002	−0.105
Ln(1+Cul) * Ln(1+EFId)			−0.092 ***	−0.147 ***
LnGDP	0.003 *	0.000	0.002	0.001
LnCGDP	0.014 **	0.010	0.010 **	0.010 **
LnGIST	−0.019 ***	−0.028 ***	−0.021 ***	−0.021 ***
LnInves		−0.014	−0.005	0.005
LnJudi		−0.020 *	−0.021 **	−0.012
Contig	0.018 ***	0.030 ***	0.020 ***	0.010 ***
Comlang	0.021 ***		−0.004	−0.023
Comrelig	−0.004 *		−0.001	0.000
Comleg	0.011 ***		0.006 *	0.006 **
Constant	−0.115	−0.009	−0.080	−0.104 *
Year	Yes	Yes	Yes	Yes
N	420	420	420	420
Adjusted R^2^	0.377	0.319	0.283	0.238

Note: Asterisks indicate significance levels: * *p* < 0.1, ** *p* < 0.05, *** *p* < 0.01.

**Table 5:** The robustness results.

Variable	Greenfield Investment (KS)	Greenfield Investment (EU)	M&A (KS)	M&A (EU)
Ln(1+Cul)	0.053 **	0.086 ***	0.063 **	0.093 ***
Ln(1+WGId)	0.038 *	0.099 *	0.043 *	0.117 *
Ln(1+EFId)	0.049 **	0.088 **	0.045 **	0.079 **
Ln(1+Cul) * Ln(1+WGId)	−0.001	−0.099	−0.003	−0.109
Ln(1+Cul) * Ln(1+EFId)	−0.081 ***	−0.135 ***	−0.098 ***	−0.156 ***
LnGDP	0.003	0.001	0.004 *	0.001
LnCGDP	0.010 **	0.010 **	0.010 **	0.010 **
LnGIST	−0.024 **	−0.024 **	−0.019 ***	−0.018 ***
LnInves	−0.002	0.002	−0.001	0.001
LnJudi	−0.017 **	−0.009	−0.019 **	−0.010
Contig	0.022 ***	0.013 ***	0.018 ***	0.009 ***
Comlang	−0.016	−0.043	−0.004	−0.022
Comrelig	−0.003	0.001	−0.002	0.001
Comleg	0.013 *	0.013 **	0.008 *	0.008 **
Constant	−0.081	−0.094 *	−0.083	−0.103 *
Year	Yes	Yes	Yes	Yes
N	420	420	420	420
Adjusted R^2^	0.272	0.229	0.276	0.231

Note: Asterisks indicate significance levels: * *p* < 0.1, ** *p* < 0.05, *** *p* < 0.01.

## Data Availability

Not applicable.
